# Acquired del(9)(p22.3) in a primary plasma cell leukemia

**DOI:** 10.1186/1755-8166-6-33

**Published:** 2013-08-28

**Authors:** Walid Al Achkar, Abdulsamad Wafa, Abdulmunim Aljapawe, Moneeb Ak Othman, Eyad Alhourani, Thomas Liehr

**Affiliations:** 1 Department of Molecular Biology and Biotechnology, Human Genetics Division, Atomic Energy Commission, P.O. Box 6091, Damascus, Syria; 2 Department of Molecular Biology and Biotechnology, Mammalians Biology Division, Atomic Energy Commission, Damascus, Syria; 3 Jena University Hospital, Institute of Human Genetics, Jena, Germany

**Keywords:** Primary plasma cell leukemia, Multiple myeloma, Del(9)(p22.3), Array-proven multicolor banding, Prognostic factors

## Abstract

**Background:**

Plasma cell leukemia (PCL) is a rare lymphoproliferative disorder, accounting for 1-2% of all plasma cell neoplasms, characterized by the presence of >2 × 10^9^/l of plasma cells circulating in the peripheral blood, and exists in two forms: primary PCL (pPCL, 60% of the cases), and secondary PCL (sPCL), the latter being a leukemic transformation in patients with a previously diagnosed multiple myeloma. PCL is an aggressive disease with poor prognosis and a short median survival of 7 months.

**Results:**

Here, we report a pPCL case with hepatosplenomegaly, anemia, thrombocytopenia, fever, fatigue, weight loss, and plasma cell count up to 60% in peripheral blood and 80% in bone marrow. Immunophenotype was compatible with PCL. A del(9)(p22.3) was characterized using banding cytogenetics and array-proven multicolor banding (aMCB), the latter being of enormous significance to characterize breakpoint regions in detail.

**Conclusion:**

To the best of our knowledge, this is the first report of pPCL associated with a partially monosomy 9pter to 9p22.3 as a sole chromosomal abnormality.

## Background

Plasma cell leukemia (PCL) is a rare lymphoproliferative disorder, accounting for 1-2% of all plasma cell neoplasms [1]. It is defined by the presence of more than 20% plasma cells in peripheral blood and an absolute plasma cell count greater than 2 × 10^9^/L [1]. PCL can be classified into two types. The primary form (pPCL), it occurs de novo in the absence of a prior history of multiple myeloma (MM) and it presents in 60% of PCL cases, and a secondary form (sPCL) with a history of plasma cell myeloma (PCM) which have progressed to a leukemic phase [1,2].

Clinically, patients with primary PCL have a higher incidence of hepatosplenomegaly and lymphadenopathy, and less lytic bone lesions [3]. Prognostic indexes such as B2-microglobulin, plasma cells in S-phase, proteinuria, calcium levels, LDH and renal function usually are found to be significantly higher in PCL than in MM [2].

PCL is highly aggressive, with unsatisfactory responses to therapy, a poor prognosis, and a median survival of 7 months [2]. Due to the low frequency of this entity, most publications on PCL are based on case reports, only a few series exist with more than 20 patients in the medical literature, and difficulties remain in defining the biological, clinical, and prognostic features of the disease [2,3].

Here we reported a new case of pPCL with a del(9)(p22.3), which was characterized by array-proven multicolor banding (aMCB); also the case had an immunophenotype result consistent with PCL.

## Methods

### Case report

A 57-year-old male was diagnosed as suffering from pPCL. Hepatosplenomegaly, anemia, thrombocytopenia, fever, fatigue, and weight loss were the indicative symptoms. His hematologic parameters were: white blood cells (WBC) of 2.7 × 10^9^/l with 60% plasma cells, red blood cell (RBC) count was 3.48 × 10^6^/mm^3^, hemoglobin level was 10.6 g/dl and the platelet count was 67 × 10^9^/l. The serum creatinine was 1.07 mg/100 ml (normal level up to 1.5 mg/100 ml); calcium, 8.5 mg/100 ml (normal level up to 10.2 mg/100 ml); serum lactate dehydrogenase (LDH) level was 662 U/l (normal level up to 480 U/l); serum aspartate aminotrasferase (AST) level was 42 U/l (normal up to 40 U/l); and serum ß2-microglobulin level was 4.12 mg/l (normal level up to 2.3 mg/l). Total serum protein was within normal range at 7.3 gm/dl (normal value 6-8.5 gm/dl) but serum albumin was 3.4 gm/dl (normal value 3.8-5 gm/dl); serum IgG level was 812 mg/dl (700-1600 mg/dl); serum IgA level was 140 gm/dl (70-400 mg/dl); and serum IgD level was 48.9 mg/dl (40-230 mg/dl). Bone marrow aspiration revealed an 80% of plasma cells. No treatment had been administered prior to the test.

All human studies have been approved by the ethics committee of the Atomic Energy Commission, Damascus, Syria and have therefore been performed in accordance with the ethical standards laid down in the 1964 Declaration of Helsinki and its later amendments. The patient gave his informed consent prior to its inclusion in this study.

### Chromosome analysis

Chromosome analysis using GTG-banding was performed according to standard procedure prior chemotherapeutic treatment [4]. A total of 20 metaphase cells derived from unstimulated bone marrow culture were analyzed. Karyotypes were described according to the International System for Human Cytogenetic Nomenclature (ISCN) [5].

### Molecular cytogenetics

FISH using a chromosome-9-specific aMCB probe set based on microdissection derived region-specific libraries was done as previously reported [4]. Also paints for whole chromosomes 11 and 14 (wcp-probes) were applied (MetaSystems, Altlussheim, Germany) according to manufacturer’s instructions. A total of 20 metaphase spreads were analyzed, each using a fluorescence microscope (AxioImager.Z1 mot, Zeiss) equipped with appropriate filter sets to discriminate between a maximum of five fluorochromes plus the counterstain DAPI (4',6-diamino-2-phenylindole). Image capturing and processing were carried out using an ISIS imaging system (MetaSystems, Altlussheim, Germany).

### Flow cytometric immunophenotype

Immunophenotyping was performed according to [6]. Manufacturer’s instructions (BD Biosciences) were also considered. Flow cytometric analysis was performed using a general panel of fluorescent antibodies against the following antigens typical for different cell lineages and cell types: CD1a, CD2, CD3, CD4, CD5, CD8, CD10, CD11b, CD11c, CD13, CD14, CD15, CD16, CD19, CD20, CD22, CD23, CD32, CD33, CD34, CD38, CD41a, CD45, CD56, CD57, CD64, CD103, CD117, CD123, CD138, CD209, CD235a and CD243; In addition to antibodies to Kappa and Lambda light Chains, IgD, sIgM, and HLADr. All antibodies purchased from BD Biosciences. Samples analyzed on a BD FACSCalibur™ flow cytometer. Autofluorescence, viability, and isotype controls were included. Flow cytometric data acquisition and analysis conducted by BD Cellquest™ Pro software. Interpretations of flow cytometric results were according to [7].

## Results

Pathology results showed that bone marrow specimen had very high cellularity. Bone marrow consisted of a morphologically homogenous abnormal cell population having plasma cell features.

**Figure 1:**
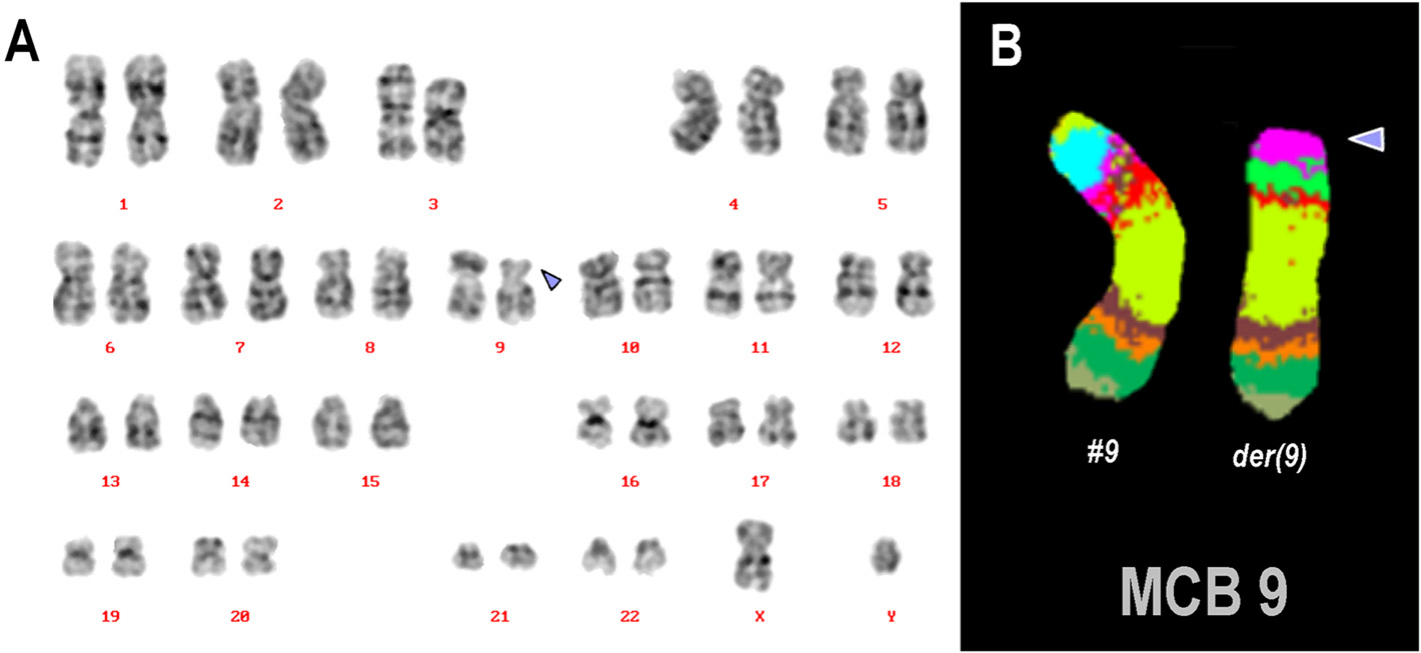
**Cytogenetics and molecular cytogenetics results. (A)**. GTG-banding revealed a del(9)(p22.3). The derivative chromosome is highlighted by arrow head. **(B)**. The application of aMCB(9) characterized the del(9)(p22.3) comprehensively. Abbreviations: # = chromosome; der = derivative chromosome.

Prior to chemotherapy treatment banding in cytogenetics revealed a karyotype 46,XY[12]/46,XY,del(9)(p?)[8] (Figure 1A). An aMCB using a specific probe for chromosome 9 revealed a deletion a part of short arm of chromosome 9 was present (Figure 1B). The final karyotype was determined as: 46,XY[12]/46,XY,del(9)(p22.3)[8] as a t(11;14)(q13;q32) could be excluded using wcp probes (results not shown).

The abnormal cell population showed the following immunophenotype: CD45^+dim^(80%), HLADr^+^(78%), CD10^+^(80%), CD38^+bright^ (88%), CD22^+^(64%), CD117^+^(73%), CD19^+^(80%), CD20^+^(64%), CD32^+^ (63%) and expressed CD13 (16%), CD138 (30%), CD15 (24%), sIgM (52%) heterogeneously. This population negatively reacted with antibodies to CD34, CD243, CD16, CD56, CD2 CD3, CD5, CD23, CD11c and CD103. CD138 expression was heterogeneous; although abnormal cell population represented 80% of all cells in lymphocytes gate, only 30% of those were CD138 positive. Expression of surface IgM was dim and heterogeneous; only 52% of abnormal cells in the lymphocytes gate reacted positively with mouse anti-human IgM (isotype IgG1-Kappa, clone G20-127). Overall, this immunophenotype was mostly consistent with PCL [7].

## Discussion

PCL is a rare entity; we described a case of pPCL with a de novo del(9)(p22.3). To the best of our knowledge, the present case is the only one ever seen with this kind of aberration [8].

According to the literature, the plasma cells have a characteristic phenotype: Strong expression of CD38 and CD19 and weak expression of CD56. In contrast to normal plasma cells, myeloma cells are often immature and may have the plasmablastic appearance [9]. While the expression of CD19 is usually negative, myeloma cells express CD56 antigen strongly along with CD38 [10].

Plasma cells in PCL display a more immature phenotype than in MM, as to be assessed by expression of CD20 antigen, the latter being usually absent in MM [11]. In addition plasma cells in PCL frequently lack CD56 antigen, which has been considered important in anchoring plasma cells to bone marrow stroma [12]. Immunophenotypic expression is similar in PCL and MM for CD38, CD138, CD2, CD3, CD6, CD10, CD13 and CD15 [3]. The phenotypic differences do not allow a complete discrimination between PCL and MM, but help to explain the differences in survival, since CD56 expression has been associated with good prognosis while CD20 expression has been associated with a shorter survival [13].

Recent attempts at genetic and molecular profiling of PCL have provided important clues to its biology. Using karyotyping, cytogenetic abnormalities have been identified in over 70% of PCL patients. Hypodiploidy and complex karyotypes with multiple numerical and structural abnormalities involving chromosome 1, 13, and 14 have been identified in a significant number of PCL cases [14]. Molecular genetic abnormalities including del(13q), del(17p), t(11;14), t(4;14), t(4;16) del(1)(p21), and 1q21-amplifications have been reported [1,14].

Primary PCL has a more aggressive course high frequency of extramedullary involvement (liver, spleen, lymph nodes, extra osseous plasmacytomas etc.), thrombocytopenia, anemia, hypercalcemia and impaired renal function [3]. PCL displays multiple adverse prognostic indicators at presentation such as elevated LDH, elevated ß2-microglobulin, hypercalcemia, high percentage of Bence-Jones proteinemia, and extramedullary involvement [13].

The prognosis for pPCL is unfavorable, with a median overall survival of 7 months [2]. In preceding reports, the unfavorable prognostic risk factors were a lack of response to treatment, the increased levels of LDH, and the peripheral plasma cell count [15]. Deletions in 9p are found in about 9% of cases of adult acute lymphoblastic leukemia. The prognostic impact of deletions in 9p has been discussed controversial [16].

In conclusion, we reported a de novo case of pPCL with a new cytogenetic abnormality, a partial monosomy 9pter to 9p22.3. Overall this finding may be regarded as unfavorable prognostic parameter in PCL, however, it could not be confirmed in the present case, as the patient was lost during follow-up.

## Competing interests

The authors declare that they have no competing interests.

## Authors’ contributions

WA-A, AA and AW provided the case and/or did primary cytogenetic and main part of the FISH-tests and flowcytometry; MAKO,EA and TL did detailed FISH studies. WA drafted the paper and all authors read and approved the final manuscript.
